# The dopaminergic system in neurodevelopment: preclinical models of neurodevelopmental disorders and susceptibility to neurodegeneration

**DOI:** 10.3389/fncel.2026.1782731

**Published:** 2026-02-23

**Authors:** Michele Santoni, Andrea Mastio, Marco Pistis, Claudia Sagheddu

**Affiliations:** 1 Department of Biomedical Sciences, University of Cagliari, Cagliari, Italy; 2 Unit of Clinical Pharmacology, University Hospital, Cagliari, Italy; 3 Cagliari Unit, Neuroscience Institute, National Research Council, Cagliari, Italy

**Keywords:** Alzheimer’s disease (AD), attention deficit hyperactivity disorder (ADHD), autism spectrum disorders (ASD), maternal immune activation (MIA), mesocorticolimbic system, Parkinson disease (PD), schizophrenia, ventral tegmental area (VTA)

## Abstract

The dopaminergic system plays a pivotal role in neurodevelopment, guiding the formation and refinement of neural circuits underlying salience attribution, cognition, reward and aversion. Its maturation extends from prenatal life through adolescence and may be influenced by genetic and environmental factors. Evidence from preclinical models suggests that perturbations during these sensitive windows may alter neurodevelopmental trajectories toward maladaptive outcomes, increasing vulnerability to neurodevelopmental disorders. This mini-review synthesizes findings from animal models to examine how physiological dopaminergic maturation might be shaped by genetic, as well as environmental, factors. We discussed maternal immune activation, prenatal cannabis exposure, and genetic models directly targeting dopaminergic function, all of which underscore the critical role of dopamine dysregulation in shaping neurodevelopmental outcomes. Beyond neurodevelopmental disorders, we extend this framework to newly emerging evidence concerning how early-life dopaminergic perturbations may influence dopamine system resilience and predispose individuals to accelerated cognitive decline and neurodegenerative disorders. Midbrain dopamine neurons exhibit intrinsic vulnerabilities that may render them especially sensitive to cumulative developmental and aging-related stressors and may serve as early predictors of disease. Finally, we discuss the therapeutic implications, emphasizing the limited mechanistic innovation in current pharmacological treatments and the growing need to target upstream or convergent developmental mechanisms in order to modify disease trajectories before overt dopaminergic dysfunction becomes established.

## 1 Introduction

The dopaminergic system plays a pivotal role in neurodevelopment, guiding the formation and refinement of neural circuits underlying salience attribution, cognition, reward and aversion (Klein et al., 2019). Its maturation extends from prenatal life through adolescence and may be influenced by genetic and environmental factors (Padmanabhan and Luna, 2014). Dopamine receptors expression emerges early in development and undergoes dynamic changes throughout postnatal life, reaching stabilization during adulthood (Garritsen et al., 2023). Due to the longitudinal period of maturation, perturbations occurring at different stages can increase the risk for the emergence of neurodevelopmental disorders (Ijomone et al., 2025). Preclinical research has been instrumental in elucidating how genetic and environmental insults during these critical windows can alter the dopaminergic system, leading to trajectories associated with neurodevelopmental disorders such as autism spectrum disorder and schizophrenia (Cai et al., 2021; Han et al., 2021). In this context, genetic models carrying mutations in key synaptic and regulatory genes have provided valuable insights into how alterations in synaptic plasticity converge on dopaminergic dysfunction, offering mechanistic insights to neurodevelopmental disorders (Contestabile et al., 2025; Parenti et al., 2020). Moreover, substantial evidence indicates that environmental insults during gestation, such as infections, maternal stress, and exposure to toxic agents might interfere with fetal brain development, thereby heightening susceptibility to disease in later life (Shook et al., 2022). Exposure to inflammatory cytokines during gestation may affect dopaminergic pathways in the offspring, altering neuronal excitability, receptor expression, and behavioral responses to reward- or stress-related stimuli (Purves-Tyson et al., 2021; Santoni et al., 2023; Santoni et al., 2022; Weber-Stadlbauer et al., 2021). These changes co-occur with disruptions in cytokine balance and lipid signaling, revealing a dynamic interplay between immune and dopaminergic systems that shapes brain maturation (Debs et al., 2024; Mostallino et al., 2023). In this review, we delve into current insights from preclinical models on how the physiological maturation of the dopaminergic system is influenced by genetic and/or environmental factors, from prenatal to postnatal insults, and how their convergence may underlie vulnerability or resilience to neurodevelopmental disorders. Furthermore, we extend this perspective to consider how early neurodevelopmental alterations may contribute to the emergence of neurodegenerative diseases.

## 2 Developmental trajectories of the dopamine system: critical periods and environmental modulation

**Figure 1:**
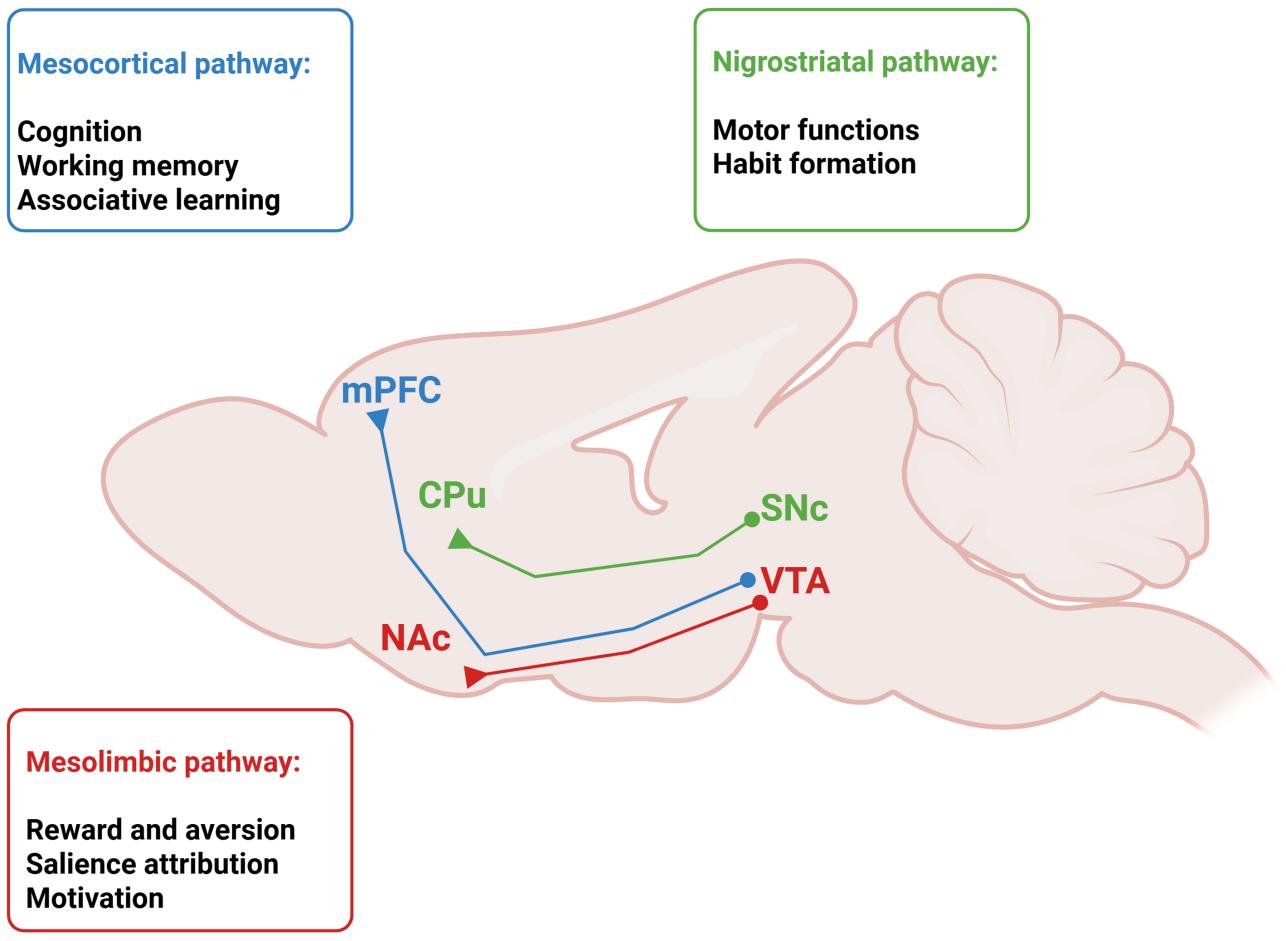
Schematic representation of the mesocorticolimbic system in the rodent brain dissected into mesocortical and mesolimbic pathways, and of the nigrostriatal pathway, with their main functions. CPu, caudate putamen; NAc, nucleus accumbens; mPFC, medial prefrontal cortex; SNc, substantia nigra pars compacta; VTA, ventral tegmental area. Created with Biorender.

Neurodevelopment represents a crucial stage in the formation of adult behavior and may create conditions for vulnerability to disease in adulthood. Although genetic factors contribute significantly to the risk of psychiatric disorders, a growing body of evidence suggests that environmental factors play a substantial role, particularly during the embryonic period. Over the past two decades, dopamine has emerged as a key in brain development, coordinating neuronal differentiation and synaptic refinement (Cai et al., 2021). The dopaminergic system proceeds through tightly regulated stages, beginning in early embryogenesis and extending into young adulthood. Here, mesencephalic dopamine progenitors arise in the ventral midbrain around embryonic day (E) 9.5, with a peak between E12 and E13, when the substantia nigra pars compacta (SNc) and ventral tegmental area (VTA) form (Islam et al., 2021), while tyrosine hydroxylase (TH), the rate-limiting enzyme in dopamine synthesis, begins to be expressed between E10 and E10.5 (Dumas and Wallén-Mackenzie, 2019). Environmental perturbations during gestation, including stress, infections, and inflammation, might influence dopaminergic development increasing susceptibility to neuropsychiatric disorders, including schizophrenia and autism spectrum disorders (Channer et al., 2023). Additionally, altered neurogenesis has been associated with an increased risk of Parkinson’s disease (von Linstow et al., 2020), and accumulating evidence supports the hypothesis of an intrinsic neurodevelopmental vulnerability of dopaminergic neurons, encompassing both the VTA and the SNc, which may underlie susceptibility to multiple neurodegenerative disorders, including Alzheimer’s disease (Krashia et al., 2019). Dopaminergic innervation of the medial prefrontal cortex (mPFC) continues to mature during early postnatal development, with fibers gradually extending from deep to superficial cortical layers until postnatal day 21 (Islam et al., 2021). Beyond early postnatal development, adolescence constitutes a major sensitive period for dopaminergic maturation, particularly within mesocorticolimbic circuits (Figure 1). This prolonged developmental trajectory results in an extended window of vulnerability, during which environmental challenges such as stress exposure or substance abuse can interfere circuit refinement and increase the risk of psychiatric disorders later in life (Cattaneo et al., 2024; Patel et al., 2021). In rodents, adolescent cannabinoid exposure impairs PFC network function (Miller et al., 2019) and is associated with subcortical dopaminergic hyperactivity, particularly within the VTA, leading to cognitive and affective phenotypes reminiscent of schizophrenia (Renard et al., 2018). All drugs associated with substance use disorders, exert their reinforcing effects by modulating brain dopamine signaling (Koob and Volkow, 2016). Cannabis and its psychoactive constituent, Δ^9^-tetrahydrocannabinol (THC), induce transient elevations in dopamine transmission, an effect that is thought to result primarily from the disinhibition of midbrain dopaminergic neurons (Peters et al., 2021). The endocannabinoid–dopamine interaction is essential for the encoding of reward prediction and teaching signals by midbrain dopamine neurons (Luján et al., 2023; Sagheddu et al., 2015). Moreover, early adolescent stress induces a hyperdopaminergic state characterized by increased population activity of VTA, but not SNc, dopaminergic neurons and an enhanced locomotor response to amphetamine (Gomes et al., 2020). Accordingly, the “two-hit hypothesis” posits that initial developmental insults, such as prenatal inflammation or genetic vulnerability, sensitize the dopaminergic system, while adolescent stress or environmental insults act as a second hit that unmasks latent dysfunctions and precipitates neuropsychiatric phenotypes (Guerrin et al., 2021; Santoni and Pistis, 2024).

### 2.1 Dysfunctions of the dopamine system in animal models of neurodevelopmental disorders

Animal models have played a central role in elucidating mechanisms regulating the development of the dopaminergic system, and its involvement in neurodevelopmental disorders, particularly schizophrenia and autism spectrum disorders (Damianidou et al., 2022; Gullapalli et al., 2025; Smucny et al., 2023). Although they do not reproduce the complexity of human phenotypes, they offer an experimental context that allows for causal analysis of the impact of genetic and environmental factors on dopamine circuits maturation and functions. In terms of brain development, the time window between the last week of pregnancy and the first three days after birth in rodents is generally regarded as corresponding to a developmental stage spanning the late second and early third trimester of human gestation (Semple et al., 2013). Accordingly, several prenatal experimental paradigms have been developed to model early-life risk factors. Among these, maternal immune activation (MIA) models have been shown to induce behavioral and neurochemical traits of neurodevelopmental disorders in the offspring (Bauman and Van de Water, 2020; Hanson et al., 2022; Santoni and Pistis, 2024; Capellán et al., 2023). Activation of the maternal immune system and the resulting pro-inflammatory response represent a key mechanism in the dysregulation of dopaminergic development. Several studies have reported that sensorimotor gating deficits are not detectable during adolescence but become evident in adulthood (Ding et al., 2019; Santoni et al., 2023). Consistently, converging evidence indicates that dopaminergic signaling is altered at adult stages rather than during earlier developmental periods. At the circuit level, the offspring display marked neurobiological changes within brain regions critically implicated in the pathophysiology of neurodevelopmental disorders, including the VTA, PFC, nucleus accumbens (NAc), and hippocampus (Ciano Albanese et al., 2025; Mueller et al., 2021). MIA male offspring exhibit marked alterations in VTA dopamine neuron activity, accompanied by increased dopamine release in the NAc, supporting the presence of a hyperdopaminergic state emerging in adulthood (Luchicchi et al., 2016). Interventions aimed at reducing gestational inflammation have proven effective in attenuating the neurobehavioral deficits induced by MIA (De Felice et al., 2018; Mostallino et al., 2023; Romero-Miguel et al., 2023). In line with this framework, prenatal disruption of dopamine system development through exposure to THC leads to long-lasting, sex-dependent alterations of mesolimbic dopamine function, resulting in a hyperdopaminergic and psychotic-like endophenotype in male offspring (Frau et al., 2019; Sagheddu et al., 2021; Traccis et al., 2021). Here, prenatal THC induces an aberrant dopaminergic function *in vivo* in male offspring that show a reduced population activity of VTA dopamine neurons and an increased sensitivity to dopamine D2-receptor activation, along with THC-induced larger increase of extracellular dopamine in the NAc (Sagheddu et al., 2021). In contrast, female offspring show a resilient phenotype associated with preserved dopamine function in the VTA, in agreement with no difference found in THC-induced extracellular dopamine in the NAc (Traccis et al., 2021). Together with prenatal immune challenges, genetic models directly targeting dopaminergic function underscore their role in shaping neurodevelopmental outcomes. Hence, genetic models lacking dopamine transporter (DAT) such as DAT-KO rats, demonstrate a increased dopaminergic state with hyperactivity and compulsive stereotypies alongside reduced reward sensitivity and impaired decision-making across development, and altered motivated behavior (Adinolfi et al., 2019; Cinque et al., 2018). The sustained hyperdopaminergia in DAT-KO rats, as measured by neurochemical experiments in the NAc (Sanna et al., 2020), triggers significant downstream alterations in neurotrophic signaling pathways that are crucial for synaptic plasticity. The DAT-KO rat model displays predictive validity (i.e., the ability of the model to predict clinical treatment responses) through its response to psychostimulant medications. While drugs like amphetamine and methylphenidate induce hyperactivity in wild-type animals, they produce a paradoxical behavioral suppression of hyperactivity in DAT-KO rats, significantly reducing their locomotor activity. This response mirrors the therapeutic action of these drugs in individuals with attention-deficit/hyperactivity disorder (ADHD), providing evidence for the model’s clinical relevance (Leo et al., 2018). Taken together, these findings suggest that persistent dopaminergic dysregulation originating early in development may not be restricted to neurodevelopmental phenotypes but could also shape long-term susceptibility to brain dysfunction across the lifespan.

## 3 From neurodevelopmental disorders to neurodegeneration: a lifespan dopaminergic vulnerability framework

While the association between neurodevelopmental alterations and psychiatric disorders is extensively studied, only very recently the perinatal developmental window is being recognized as substrate for long-term vulnerabilities that predispose individuals to neurodegeneration in late life. Same early-life genetic, epigenetic and environmental factors that shape brain maturation and function, influence vulnerability and/or resilience to aging-related stressors. This developmental origin framework could explain why individuals with subtle early-life alterations may exhibit accelerated decline when aging introduces cellular and molecular perturbations, such as DNA damage responses, epigenetic dysregulation, and synaptic dysfunction (Iii et al., 2023; Long, 2024). Midbrain dopamine neurons of the SNc are characterized by peculiar morphological and biochemical features, including long axons and extensive axonal arborization, sustained Ca^2+^ influx, dopamine oxidation, and high metabolic demand (Pacelli et al., 2015; Surmeier, 2018). These features, while essential for motor control, render them particularly vulnerable to oxidative stress and mitochondrial dysfunction, ultimately favoring age-related damages and particularly α-synuclein pathology (Naoi et al., 2024; Palmas et al., 2022). Variations in the number of dopamine neurons across neurogenesis and developmental stages have been linked to the probability and temporal onset of Parkinson’s disease (von Linstow et al., 2020). Recent studies highlight that neuronal vulnerability is not uniform but depends on developmental subtypes, whereby distinct neuronal populations exhibit earlier degeneration compared to others, possibly due to mechanisms involving DAT degradation by autophagy (Harraz, 2023). Indeed, misregulation of developmental cellular and molecular functions such as axonal guidance, trophic signaling or DAT expression can further reduce resilience, setting the stage for Parkinson’s disease.

Despite the link between intrinsic vulnerability of dopamine neuron and neurodegeneration appears more evident for SNc neurodegneration in Parkinson’s disease, increasing evidence correlates the VTA and Alzheimer’s disease (Siguier et al., 2025; Long, 2024; Sharif et al., 2025). While the dopamine system is extensively studied in neurodevelopmental and associated psychiatric conditions, its involvement in Alzheimer’s disease has long remained poorly understood and largely neglected in research. Impairments in the VTA are recognized both in animal models and in humans since the very initial stages of disease (La Barbera et al., 2025; Pilotto et al., 2025; Spoleti et al., 2024). Consistently, in aged rats preserved/impaired VTA dopamine cell function is associated with high/low performance, respectively (Sagheddu et al., 2024), together with increased cognitive and behavioral outcome following selective enhancement of dopamine neurotrasmission (Lubec et al., 2023; Lubec et al., 2021).

Emerging evidence indicates that the dopamine system can be involved since neurodevelopment to predispose individuals to earlier onset and accelerated progression of Alzheimer’s disease. Disruptions in dopamine signaling have been identified as early markers of cognitive decline, suggesting possible involvement of dopaminergic neurodevelopmental dysfunction toward neurodegeneration (Zaccone et al., 2025). Complementary evidence from imaging studies of the VTA underscores its importance as a diagnostic and therapeutic target, as early dopaminergic dysfunction in this region is associated with neuropsychiatric symptoms and accelerated disease progression (Krashia et al., 2022). Altogether, these data suggest that maladaptive developmental tuning of dopamine system increases susceptibility to neurodegeneration in late life, highlighting the importance of examining neurodevelopmental steps within a longitudinal framework. Further studies are needed to investigate molecular mechanisms and the causal relationship between neurodevelopmental alterations of dopamine system and dementias.

## 4 Discussion

The physiological maturation of the dopamine system is crucial in shaping developmental trajectories relevant to neurodevelopmental disorders such as schizophrenia and autism spectrum disorders (Ijomone et al., 2025). Its development is characterized by tightly regulated stages extending from early embryonic life into young adulthood (Larsen et al., 2020). In this context, gestational environmental challenges, such as stress, infection, and inflammation, may influence the development of dopaminergic circuits (Channer et al., 2023). Epidemiological evidence indicates that exposure to elevated maternal proinflammatory cytokine levels during early pregnancy may contribute to an increased risk of psychosis later in life (Allswede et al., 2020). Beyond the proposed involvement of altered mesolimbic dopamine function, morphological and neurochemical changes within the prefrontal dopaminergic system are also likely to contribute to behavioral and cognitive impairments observed in adult offspring born to immune-challenged mothers (Perez-Palomar et al., 2023). In addition to models of gestational immune activation, prenatal exposure THC has also been examined as an early-life risk factor affecting dopaminergic system development. Dysregulated dopamine transmission and associated cortical dysfunctions have been proposed as mechanisms underlying several behavioral outcomes reported in human studies (Bara et al., 2018; Solmi et al., 2023). Across different animal models, prenatal cannabis exposure has been shown to exert negative and long-lasting effects on dopamine signaling, leading to alterations that partially overlap with those observed following gestational immune challenges (Frau et al., 2019; Hurd et al., 2019). Prenatal THC exposure has been associated with alterations in excitation-to-inhibition balance in the VTA, changes in dopamine neuron activity, and increased sensitivity of dopamine D2 receptors, together with modifications in the expression of genes encoding dopaminergic receptors in target regions (Sagheddu et al., 2021; Traccis et al., 2021). These neurobiological alterations may contribute to deficits in cognitive performance, attentional control, and impulse regulation observed in the offspring of mothers who consumed cannabis during pregnancy (Bara et al., 2018; Solmi et al., 2023). MIA and prenatal THC exposure models indicate that different early-life challenges may share overlapping neurodevelopmental processes. Consistently, alterations in endocannabinoid-mediated synaptic plasticity within mesolimbic regions have been reported across models, suggesting the involvement of convergent mechanisms in neurodevelopmental impairment (Santoni and Pistis, 2024; Solinas and Melis, 2024). In line with the convergence observed across prenatal immune activation and prenatal THC exposure models, genetic approaches directly targeting dopaminergic regulation further support the involvement of shared developmental mechanisms underlying dopaminergic vulnerability. Thus, genetic models of dopamine transporter dysfunction, such as DAT-KO rats, show that impaired dopamine clearance leads to a persistent hyperdopaminergic state, coupled with hyperactivity, stereotypies and reduced reward sensitivity (Adinolfi et al., 2019; Cinque et al., 2018). It should be acknowledged that a broad range of transgenic animal models of neurodevelopmental disorders have provided valuable insights into disease mechanisms at the level of specific neural circuits and cell types. These include well-established models of conditions such as Rett syndrome and Fragile X syndrome. A detailed discussion of these models is beyond the scope of this review (for excellent reviews see Damianidou et al., 2022; Premoli et al., 2021). This perspective opens the question of whether early dopaminergic perturbations may modulate susceptibility to later-life neurodegenerative processes. Emerging evidence points to a plausible involvement of dopaminergic systems from early developmental stages in shaping susceptibility to neurodegenerative diseases, with alterations in acetylcholine and dopamine signaling reported as early correlate of cognitive decline. Indeed, the dopaminergic system undergoes multiple changes during normal or impaired aging (Krashia et al., 2022; Sagheddu et al., 2024). These include altered electrical activity, reduced dopamine release from mesocorticolimbic terminals, decreased expression of dopamine receptors, particularly D2 subtype, and lower DAT expression in regions such as the NAc, putamen, hippocampus, and PFC (Martorana and Koch, 2014; Norrara et al., 2018). Consistently, dopamine neuron degeneration in the VTA leads to hippocampal hyperexcitability in experimental Alzheimer’s disease (Spoleti et al., 2024). These observations raise interest in therapeutic strategies aimed at preserving or modulating dopaminergic function during the mild cognitive impairment stage, prior to overt neurodegenerative progression. At the same time, the lack of pharmacological interventions capable of effectively mitigating dopaminergic system alterations across both neurodevelopmental and neurodegenerative disorders highlights the substantial complexity of these conditions. In the field of dementias such as mild cognitive impairments and Alzheimer’s disease, current pharmacological approaches remain largely based on acetylcholinesterase inhibitors, with limited disease-modifying efficacy despite the recent clinical introduction of monoclonal antibodies as anti-amyloid immunotherapies (Sadruddin et al., 2025). In ADHD, pharmacological treatment continues to rely primarily on DAT inhibition, underscoring the relative stagnation of mechanistic innovation in this area (Veronesi et al., 2024). For readers interested in an overview of the latest advances in novel therapeutic approaches for neurodevelopmental and neurodegenerative disorders, we refer to several excellent recent reviews highlighting emerging strategies and targets (Baribeau and Anagnostou, 2022; Coyle and Paul, 2026). Within this framework, several upstream mechanisms emerge as plausible pharmacological targets. These include modulation of neurotransmitter systems (i.e., adrenergic, muscarinic, GABAergic, glutamatergic; Tobin, 2024; Tang et al., 2021; Frau et al., 2022; Parent and Niswender, 2024; Devoto et al., 2020), immune processes and inflammation, as well as interventions on endocannabinoid-gut-brain axis (Campanale et al., 2025). While most current interventions are applied after symptom onset, it would be desirable to move beyond late symptomatic treatment toward preventive and early-stage strategies, consistent with a precision-medicine approach aimed at limiting alterations that may impair dopaminergic system function and thereby affect neurodevelopmental trajectories, as well as reduce vulnerability to neurodegenerative processes across the lifespan.
